# Supplementary material for: Effect of airway clearance therapies on mucociliary clearance in adults with cystic fibrosis: A randomized controlled trial
Source: PLoS One. 2022 May 20;17(5):e0268622. doi: 10.1371/journal.pone.0268622 (PMC9122229; doi:10.1371/journal.pone.0268622)
Supplement: S4 File — Study protocol prepared a priori, as reviewed by the institutional board. (PDF) [file pone.0268622.s005.pdf]

Researching the Effects of Airway Clearance Therapies in Cystic Fibrosis (REACT-CF)

1. Specific Aims

Mucus clearance is one of the primary innate defense mechanisms of the lungs, and impaired mucus clearance is believed to drive the development and progression of cystic fibrosis lung disease through a cycle of bacterial colonization, infection, inflammation, and mucostasis. Airway Clearance Therapies (ACTs) are a cornerstone of treatment in Cystic Fibrosis (CF), and are typically accomplished using one of a number of mechanical devices that are designed to facilitate mobilization of mucus from the airways. Despite the emphasis clinicians place on these therapies and the time patient spend performing them, little research is available to validate the efficacy of any specific device or technique; even less research exists on the comparative effectiveness of competing devices.

The biology underlying the transduction of physical forces applied with ACTs into effective mucus clearance has also been inadequately studied. Mechanical forces are known to stimulate release of signaling molecules (i.e. adenosine nucleotides and nitric oxide [NO]) from airway epithelia. These molecules are involved in the regulation of physiologic processes (i.e. ion transport, cilia beat frequency, and mucus secretion) that determine rates of mucociliary clearance (MCC). Until now, no studies have directly explored the linkage between these signaling molecules and *in vivo* rates of MCC. If in fact a significant relationship is discovered, the measurement of these molecules could provide simple, non-invasive biomarkers that reflect MCC rates and allow assessment of treatment effects from ACT and potentially other therapeutics.

In this study, we propose to objectively characterize the effect of ACTs that utilize different approaches by: 1) measuring MCC using gamma scintigraphy; and 2) exploring biologically plausible biomarkers that could help our understanding of the physiologic responses to ACT that facilitate mucus clearance. We also explore the effects and utility of a novel vibrating plate device as an ACT. Finally, the results of this study will used to inform the design of future trials of effectiveness of ACTs.

Within this study, we will test several hypotheses:

Hypothesis 1: *Device-assisted airway clearance therapy using OPEP, HFCWO-vest, and whole-body vibration causes a demonstrable increase in MCC and expectorated sputum volume when compared to cough alone.*

Hypothesis 2: *Airway signaling molecules, including NO and purine nucleotides (e.g. ATP, adenosine), are associated with the regulation of MCC and mediate the increase in mucus clearance that occurs following the application of mechanical stimuli from OPEP, HFCWO-vest, and whole-body vibration.*

We will test these hypotheses through these specific aims:

Specific Aim 1: Measure the effects of 4 different ACT modalities on MCC, using gamma scintigraphy.

Specific Aim 2: Measure the effects of different ACT modalities on proposed regulators of MCC, including nucleotide levels (via exhaled breath condensate collection) and exhaled NO.

Specific Aim 3: Examine associations between biomarkers of airway mechanical stimulation and measured MCC.

Specific Aim 4: Determine what study characteristics would be needed to more fully assess the effectiveness of ACTs, including sample size, study design features, and outcome measures.

2. Background And Rationale

CF and MCC

CF lung disease results from abnormal ion transport, dysfunctional regulation of airway surface liquid (ASL), and reduced mucus clearance[1-3]. Recent work has led to the development of a 2-gel model of the ASL layer where dehydration and increased osmotic pressure in the overlying secreted gel can result in volume depletion of the periciliary gel layer. This in turn may lead to adhesion of mucus plaques to airway epithelial surfaces and disruption of ciliary motion and cough-stimulated mucus clearance[4]. In CF subjects, we have shown that cilia and cough driven mucus clearance is abnormal[5,6], and that additional reduction in MCC contributes to the onset of a pulmonary exacerbation. Not surprisingly, effective airway clearance of mucus is a cornerstone of therapy and of great importance in preventing disease exacerbations and progression of airways disease.

Airway Clearance Therapies

Airway clearance has been, and continues to be, a central part of CF care. Techniques of manual chest percussion and postural drainage have largely been supplanted in adolescents and adults by the use of ACT devices, which afford patients independence. These devices, together with voluntary breathing techniques (active cycle of breathing, autogenic drainage, huff coughing) and exercise, enable individuals with CF to better expectorate tenacious airway secretions. Currently available ACT devices include pneumatic high frequency chest wall oscillatory devices, typically worn as a vest (The Vest®, AffloVest®, inCourage®, SmartVest®), a variety of oscillatory and non-oscillatory positive expiratory pressure (PEP) devices (Flutter®, Acapella®, Aerobika®, RC-Cornet®, TheraPEP®, Hydrapep®), and acoustical percussors (Vibralung). FDA clearance of these devices do not require proof of efficacy and, according to an expert review of available literature, there is insufficient evidence to assess the long-term effects of ACTs as well as inadequate evidence to support the superiority of one ACT over another[7]. Therefore, prescription of such devices is often determined by the subjective preference of each patient, based on what "feels" most effective to them, and/or provider habits and/or biases with little objective data guiding decisions.

Whole Body Vibration In Exercise And Therapy

The use of whole body vibration (WBV) via a vibrating plate was developed for use in personal fitness and recreation and not as a medical therapy or intervention. These devices consist of a large plate that vibrates at frequencies generally between 20 and 70hz and with a displacement of 1-5mm. The effect of these devices is to constantly accelerate and decelerate the whole body in an oscillating manner. This acceleration is described by the simple harmonic motion equation and is proportional to the displacement of the plate and to the square of the frequency of vibration. These variables are the settings on the device. Studies of WBV in bone density and musculoskeletal outcomes in older adults did not show benefit for acceleration <1 *g*, but other studies did show some benefit for WBV exerting higher magnitudes (1 to 8 *g*)[8-10]. A pilot study of WBV in individuals with cystic fibrosis showed an improvement in muscle strength and performance[11]. These devices have not been studied as a means of airway clearance therapy, but there is anecdotal support for their use for airway clearance by individuals with CF. Furthermore, studying their effect as an ACT allows us to test the hypothesis that high energy devices, even those not specifically engineered for airway clearance such as WBV platforms, may be as effective in ACT as those devices that were specifically engineered to affect the respiratory system.

Biomarkers of Mucus Clearance

Gamma scintigraphy has been developed as an important tool in the measurement of MCC, utilizing a technique of inhalation of an aerosolized radiotracer followed by particle detection and image processing. A standard operating procedure was developed at UNC to report resting MCC as well as cough clearance and has been used in several single and multisite studies[12]. Pharmacologic therapies in CF such as hypertonic saline[13] and ivacaftor (in patients with the G551D-mutation)[14] have yielded demonstrable improvement in clinical outcomes and MCC, whereas reduced MCC was observed at the start of a pulmonary exacerbation. Conversely, no improvement in MCC was observed with agents that have also failed to yield meaningful clinical improvements in CF (e.g. amiloride, UTP, denufosol). Together, these observations suggest that the MCC technique may have the ability to predict the clinical effectiveness of a subgroup of CF therapies.

Given the importance of MCC as an innate lung defense, it is not surprising that there are overlapping systems of local regulators that control the physiologic processes that dictate MCC rates, including ion transport and ciliary beat frequency (CBF). Extracellular adenosine 5'-triphosphate (ATP) is the endogenous ligand for $P2Y_2$ receptors, which in turn stimulates calcium activated chloride channels, inhibits the epithelial sodium channel (ENaC), and increases CBF[15,16]. ATP release in airway epithelia is very sensitive to mechanical stimulation[17], and could provide a mechanistic link between ACT or exercise and mucus clearance. Adenine nucleotide levels have been successfully measured in exhaled breath condensate (EBC) specimens from CF patients, and likely reflect airway inflammation as well[18,19].

Another airway mediator that is linked to MCC is NO. NO, via cGMP, is known to increase CBF[20,21] and may inhibit ENaC activity via the same signaling pathway[22,23]. Exhaled NO levels are significantly reduced in diseases characterized by reduced MCC (PCD and CF), and increase in response to ivacaftor in G551D-CF patients[24]. NO production may also be increased by shear stress, at least in endothelial cells[25]. Once again, like ATP, NO levels in the airway may be modulated by ACTs and impact MCC.

Slow Inhalation, Large Particle MCC Method Development

A multicenter MCC SOP was developed and tested by the UNC group[12] and has been employed in several CFF and industry funded studies (GOAL, VX661-111, AlgiPharma, PROSPECT)[14]. This method, which utilizes a relatively fast and shallow breathing pattern during isotope inhalation, emphasizes clearance from the large airways, but is less able to characterize small airway clearance. We, therefore, used funding from the MCC Consortium (CFF) to develop and test alternative methods for assessing particle clearance from the small airways. In this work, we used concepts introduced by Lindstrom et al., where the very slow inhalation of large aerosol particles (SILP) can be used to better target small airways. Slow inhalation (80 ml/sec) of 5 μm and 9 μm MMAD particles was compared to our standard SOP (5 μm MMAD with inhalation rate of 500 ml/sec) in healthy, CF and COPD subjects. First, while large airway clearance appeared unchanged (definition: whole lung clearance in first 90 minutes), the fraction of isotope that cleared between 90 minutes and 6 hours more than doubled, signifying greater small airway deposition via avoidance of alveolar deposition (Figure 1A). Second, we observed an improved ability to distinguish pathological clearance patterns in diseased subjects (Figure 1B), in contrast to our prior observation that only peripheral clearance and cough clearance appears to be impaired in CF subjects with our standard measurement protocol. Finally, we tested the relative responsiveness of the SILP method using hypertonic saline (HS), given 100 minutes, 4.5 hours, and 16 hours after isotope inhalation, to simulate a highly active and durable MCC stimulant. In these experiments, a surprisingly large acute effect of HS was observed (Figure 2A), particularly with the 9 μm particle,

and every subject was observed to be a "responder", as opposed to prior observations that only ~60% of subjects acutely respond to HS when using the traditional MCC SOP (Figure 2B). In summary, this new methodology appears to have significant advantages to the traditional MCC measurement protocol typically used in multicenter studies.

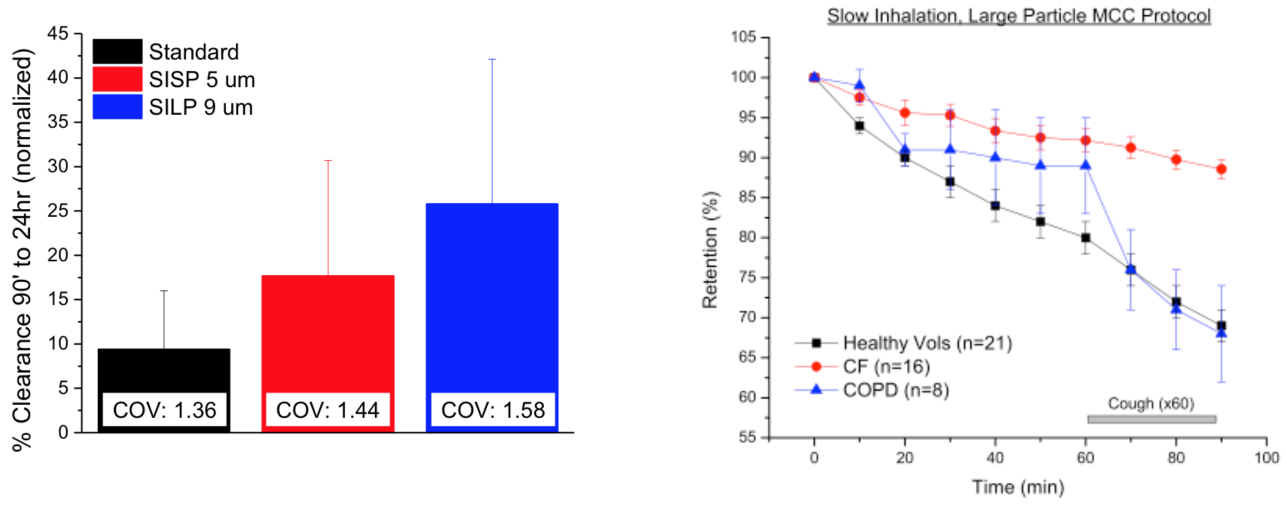

Figure 1: A) Increased small airway clearance signal with slow inhalation of 9 μm particles, vs. slow inhalation of 5 μm particles or the standard MCC protocol, (shown with COV, coefficient of variation). B) SILP MCC method distinguishes CF from health and COPD clearance patterns.

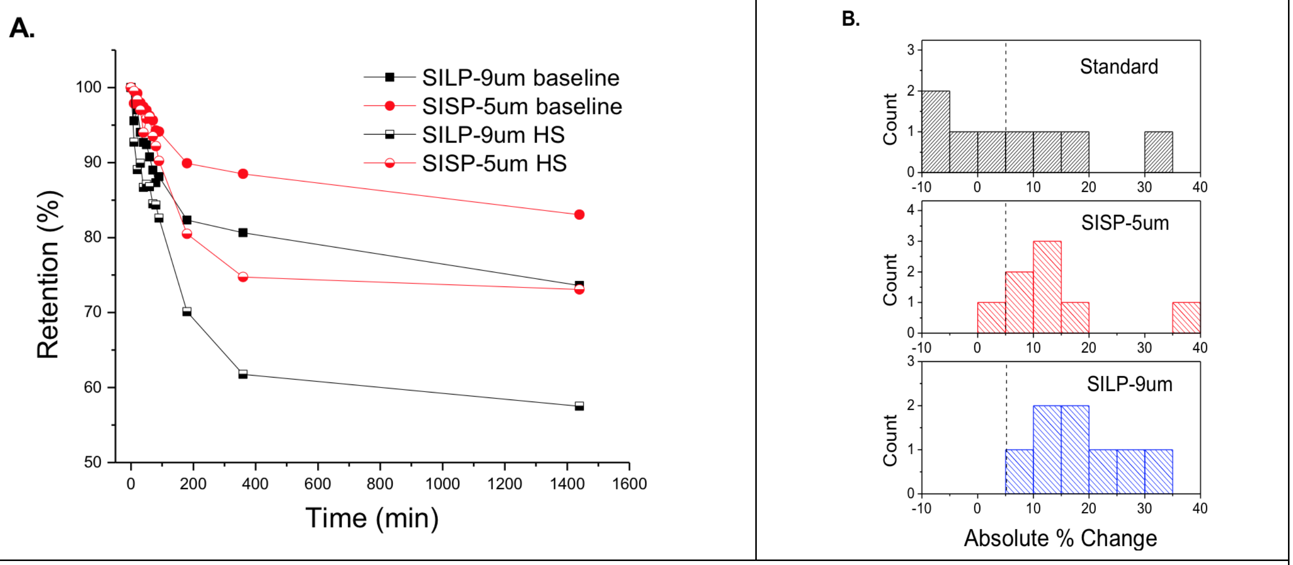

Figure 2: A) Marked treatment effect of repeated doses of HS given after SILP isotope inhalation. B) Analysis of response to HS using absolute change in clearance from baseline. HS was given after slow inhalation of 5μm and 9μm particles (lower 2 panels), and is compared to the acute response to a single HS dose using the traditional MCC SOP (historical data; Donaldson et al. NEJM 2006).

3. Significance of this research

Despite a relative paucity of evidence for its efficacy, airway clearance therapy remains a foundational component of comprehensive CF care. Well-designed randomized controlled trials examining the efficacy of specific ACTs are generally lacking, and there are few economic pressures to drive their performance. Comparison between ACTs is also hampered by the lack of outcome measures. In this study, we will employ a highly relevant outcome measure (MCC) to characterize the effect of differing ACT modalities. Further, by collecting easily obtained molecular biomarkers (FENO and EBC nucleotide levels) that have biological relevance to the mucus clearance system and are believed to transduce physical stimuli into biological signals, and correlating them to quantitative MCC measurements, we may discover broadly useful biomarkers for research into ACT effects while also broadening our understanding of the mechanisms that control mucus clearance. These biomarkers could be used to identify ACT modalities worthy of more extensive clinical testing or to "personalize" therapies to individual patients or groups of patients with specific characteristics (e.g. mild vs. severe lung disease). We will also investigate the effects of a novel device that employs high frequency, whole body vibration, but was not developed as an ACT device, to broaden our understanding the types of mechanical stimuli that successfully increase mucus clearance. The results of this pilot can be used to inform the design of future trials of effectiveness of ACTs, including sample size, study design features, and outcome measures.

4. Rationale for proposed study

We intend to carry out this pilot study to determine the effect size (and variability) of cough alone and various ACT devices on MCC, signaling molecules, and sputum volume. We will also explore the correlation between MCC and signaling molecules, and compare a novel exercise device using whole body vibration to traditional ACT methods.

5. Experimental Design and Methods:

Key inclusion criteria: Adults with confirmed diagnosis of cystic fibrosis aged 18 years and older.

Key exclusion criteria: Pregnant or lactating women, individuals with severe lung disease (FEV_1 <30%), or with exacerbations of lung disease requiring antibiotics or medication change within four weeks prior to enrolment. Individuals unable to use vest-based HFCWO, oscillatory PEP device, or who require oxygen supplementation at rest. We will also exclude individuals with significant balance or gait impairment preventing the individual from tolerating being positioned on a vibrating platform.

Study design: Ten (10) adult CF subjects will be recruited from the CF clinic at UNC, and will have a screening visit and four subsequent study visits. At Study Visit 1 huff-cough alone (control) will be performed while the order of the 3 remaining airway clearance therapy (ACT) treatments received at Study Visits 3-5 will be randomized: oscillatory PEP (positive expiratory pressure), high frequency chest wall oscillatory vest, and the vibrating plate device. Only one ACT device will be used at any given visit as adjuncts to huff-coughing, with the same cough regimen used at all study visits (Figures 3 & 4). Study visits will be at least three and no more than 21 days apart. Subjects who are prescribed hypertonic saline, dornase alfa, long-acting bronchodilators, and/or airway clearance will be asked to withold use of these agents/activities after midnight before each study visit after screening. No bronchodilators will be used as part of this study.

Methods:

Screening Visit

Following recruitment and informed consent, subjects will undergo a screening visit that includes a physical exam, medical history, medication review, and measurement of spirometry. Subjects will be allowed to continue all of their home medications during the study, but will be asked to withhold hypertonic saline, dornase alfa, and long-acting bronchodilators for 12 hours before mucociliary clearance (MCC) scans. No bronchodilators will be given.

Randomization

Baseline (huff-cough only) measurements will be obtained at Visit 2. For visits 3-5, a single ACT method will be employed at each visit, with a randomized order in which the methods are used. We will employ a Latin-square design as shown. First, the 3 periods will be randomized, followed by the sequences (A-C). This will generate a random 3 x 3 table in which each ACT appears once in each row and column. The rows of this table will serve as a block randomization schedule, which will optimize balance for a number of subjects not divisible by the number of possible sequences. Subjects will be assigned to the next row in the table after the baseline visit (repeating the sequences every 3rd subject) to ensure eligibility prior to randomization.

		**Period**		
		**1**	**2**	**3**
**Sequence**	**A**	Vest	OPEP	WBV
	**B**	WBV	Vest	OPEP
	**C**	OPEP	WBV	Vest

Study Visits

The order of procedures at each study visit is outlined in Figure 3. At each visit, subjects will be asked about intercurrent adverse events, changes in medical regimen and health status. Vital signs and a pregnancy test (if applicable) will be performed to ensure subject safety. Spirometry will not be obtained during study visits as the forced expiratory maneuver may stimulate MCC and mediator release (nitric oxide, nucleotides), and therefore may interfere with results. Exhaled breath condensate (EBC) and fraction of exhaled nitric oxide (FENO) samples will be obtained prior to the inhalation of isotope and immediately after the first ACT intervention. Procedures for biomarker collection and analysis are discussed below.

Figure 3

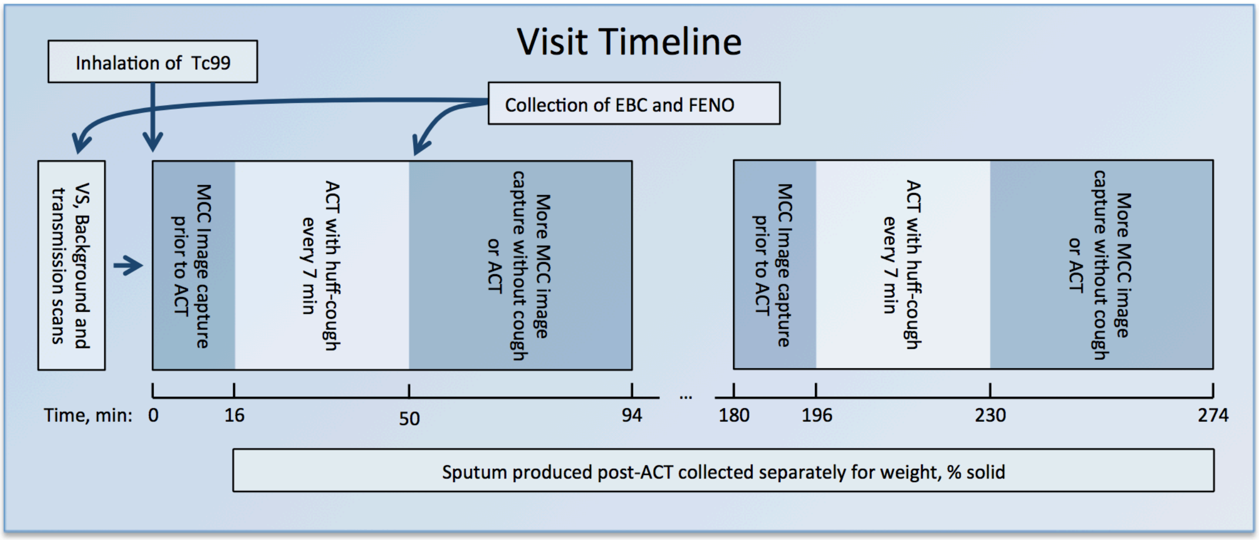

Figure 4

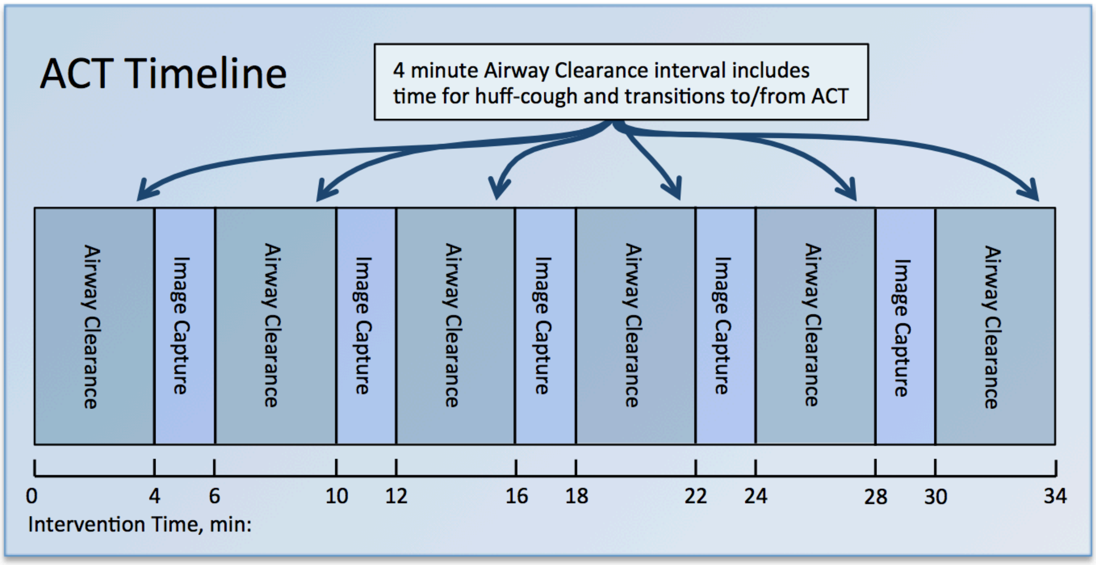

ACT Interventions

ACT interventions will be performed while capturing MCC images, as shown in Figure 4. Each 40-minute intervention period will be broken into six segments of ACT, each followed by a huff-cough maneuver. The protocol for each ACT is discussed below. Each ACT segment will be separated by two minutes of MCC image capture, which will allow quantification of particle clearance using previously described methods[6,12,26]. To perform huff-coughs, subjects will be instructed to inspire to about 75% of total lung capacity (TLC), hold inspiration for 2 seconds, and then expire forcefully to near residual volume (RV). This breathing pattern will be repeated two additional times and followed by a single, forceful cough to complete a single huff-cough maneuver. During each of the six segments of the intervention period, there will be five minutes for transitioning from image capture to ACT, completion of the ACT, performing huff-cough, and repositioning in front of gamma camera. Four minutes are allotted for ACT, transitions, and huff-cough. During the baseline visit, cough clearance will be performed during time allotted for huff-coughs but no additional interventions will take place during the 4-minute time for ACT. Immediately following the intervention period, additional EBC and FeNO will be obtained, and the subject will again be positioned in front of the camera for an additional 44 minutes of image capture.

Second Intervention Period

By repeating the visit-specific ACT intervention on the same study day (16 min and 196 minutes after isotope inhalation) we hope to identify the optimal timing of sputum volume and MCC measurements to differentiate the effects of the different ACT modalities. After the initial ACT intervention and MCC assessment, subjects will remain within the Marsico Clinical Research Center and will refrain from any inhaled medications, vigorous exercise, or additional ACT during this time. 180 minutes after inhalation of tracer, a repeat image capture and intervention period will occur identical to the first. This will conclude the study visit day.

ACT Protocols

High Frequency Chest Wall Oscillating Vest (HFCWO-Vest)

All subjects will use the The Vest® System, Model 105 (Hill-Rom, Batesville, IN). Subjects will have the adjustable vest fitted during their screening visit. The following adaption of a previously-studied protocol will be used for device settings[27]. Each treatment segment will be 4 minutes.

**Segment:**	**1**	**2**	**3**	**4**	**5**	**6**
Frequency, Hz:	8	9	10	18	19	20
Device Pressure	10*	10*	10*	6	6	6

*or highest tolerated pressure setting

Oscillating Positive Expiratory Pressure Device (OPEP Device)

Subjects will be given an Aerobika® (Monaghan Medical Corporation, Plattsburgh, NY) oscillating positive expiratory pressure system on a resistance setting of 5, the maximum resistance setting on the device. Subjects will perform 10 exhalations from near TLC to near RV through the OPEP device at a respiratory rate no faster than 10 breaths / minute to avoid acute respiratory alkalosis. The resistance setting will be decreased as necessary so subjects can comfortably perform this breathing maneuver. Subjects will perform 10 exhalations through the device during each of the six ACT segments minutes followed by one huff-cough maneuver. A member of the research team will coach subjects on proper breathing and device technique.

Vibration platform for Whole Body Vibration (WBV)

WBV will be performed for 90 seconds during each ACT segment using the PowerPlate® (Performance Health Systems, Northbrook, IL) vibration platform. Subjects will be positioned seated on the powerplate, which will rest on a custom-made wooden box 12" high, so that height of the platform is approximately that of a standard chair. Holes in the top of box engage the rubber "feet" of the PowerPlate to keep it in place. Subjects are seated with their feet on the floor and hands in their laps, sitting up as straight as possible. The "high" amplitude will be used on the device. After each segment, subjects will perform a huff-cough maneuver and return to be seated in front of the gamma camera.

Outcome Measures

Mucociliary Clearance

Background and transmission scans will be performed per the MCC Standard Operating Procedure (see attachment) to subtract background radiation levels and allow identification of lung boundaries and regions of interest[12]. The Tc-99 sulphur colloid radiotracer will be inhaled using our slow inhalation, large particle method. The subject will be positioned in front of the gamma camera, and images will be obtained for 16 minutes prior to any intervention. Sixteen minutes after particle inhalation, there will be a 40-minute intervention period with whichever ACT the subject is to receive during that study visit. Following the ACT period, there will be an additional 38 minutes of image capture. After an 86-minute break (i.e. 180 minutes after tracer inhalation), an identical MCC image capture period with ACT intervention will occur (Figure 3).

Airway Signalling Molecule Assays

<u>Exhaled Breath Condensate (EBC)</u>

EBC collections will be performed using the R-tube system. The subject will perform normal, tidal volume breathing through the chilled tube for 7 minutes, while wearing nose clips. The baseline collection will be performed at each study visit before inhaling isotope, and will be repeated immediately after the first ACT intervention at minute 56 (see Figure 3). EBC samples will be processing by adding an internal standard with isotopically labeled urea, purines, and

amino acids as previously described[18]. Following this step, the EBC sample (plus internal standard) will be lyophilized and reconstituted to achieve a 20-fold increase in concentration before analysis by mass spectrometry.

EBC concentrations of purine metabolites (AMP, adenosine, hypoxanthine) will be measured using MS methods as previously described[19,28,29]. In addition, we will utilize a recently installed AB Sciex 6500 triple quadrupole mass spectrometer available in our core center that has ~100-fold increased sensitivity relative to the Quantum Ultra mass spectrometer utilized in previous studies and should be ideal for assessing the low biomarker concentrations expected in EBC.

<u>Fraction of Exhaled Nitric Oxide (FeNO)</u>

FeNO will be measured using NIOX MINO® device by Aerocrine according to device instructions. FeNO measurement will be performed immediately before each EBC collection (i.e., before inhaling isotope and at minute 56).

Sputum Collection and coughs

All expectorated sputum from the start of the first ACT period through 274 minutes post isotope inhalation will be collected and weighed to assess total volume. Subjects will not be encouraged to cough outside of huff-cough maneuvers– rather, to cough only when naturally compelled to do so. Subjects will be encouraged to expectorate rather than swallow their sputum. This sputum will be analysed by total weight and percent solids. While MCC images are being obtained, subjects will be asked to minimize additional coughing besides the huff-coughs performed during ACT intervention period. Any additional coughs will not be controlled for, but will be recorded and evaluated as a covariate in data analyses.

Subject Evaluation of ACT

Following each treatment session, subjects will be asked to subjectively evaluate the efficacy of the device. Subjects will be to agree or disagree with the following statement using a standard Likert scale: "This ACT technique was effective for lung clearance." At the conclusion of the study, each study subject will be asked: "Which of the studied airway clearance methods would you choose to perform on a daily basis?" Responses will be limited to each of the four studied modalities.

Data management:

The data will be entered in REDCap, an electronic data capture program. The data entry forms will include edit checks to insure data quality. The discrepancies will be managed by the study coordinator. After reviewing the database for quality checks, the database will be frozen and exported at the end of the study for analysis.

Data analysis:

Agathe Ceppe, biostatistician for the UNC CF Center, has been closely involved in the study design and will be responsible for overseeing the database creation and management, as well as performing final statistical computations during data analysis.

Primary comparison: MCC will be visualized using a whole right lung clearance vs. time plot from immediately after isotope inhalation through 274 minutes post-inhalation. The area under

this curve (AUC-Clr_{274}), will be used to reflect "total clearance", and will serve as the primary outcome of this study. We will compare AUC-Clr_{274} in huff-cough alone to AUC-Clr_{274} measured with each of the ACT modalities (HFCWO-vest, OPEP device, and WBV device).

Secondary comparisons: AUC-Clr over other intervals (i.e. 94 min and 180 min), and from other lung regions of interest (i.e. peripheral and central lung zones) will be calculated and compared in similar fashion as above. Furthermore, the change in the rate of clearance (represented by the slope of the MCC curve, $d\text{Clr}/dt$) before (0-16 minutes of MCC curve; $d\text{Clr}/dt_{0\text{-}16}$) versus during/after the initial ACT intervention (16 – 56 minutes; $d\text{Clr}/dt_{16\text{-}56}$) will be used to compare the immediate effects of each ACT versus. huff coughs alone. The mean changes in EBC purines concentrations and FeNO, (before vs. after ACT) will be calculated. If these are found to be different from zero, a comparison between huff-cough alone and each of the ACT modalities will be made. Total sputum weights and % solids at baseline and after each ACT 40 minute period will be compared across each of the ACT modalities. We will also assess for a relationship between changes in EBC measurements, FeNO, and changes in MCC.

Means, standard deviations and 95% confidence intervals will be calculated for MCC parameters and all secondary outcome measures. A repeated measures model will be used to compare the primary and secondary outcome measures, with the subjects considered as random factors. For hypothesis one and three, MCC values from huff-cough alone will be compared to traditional ACT methods (HFCWO-vest and OPEP) and Whole Body Vibration to test the null hypothesis that there is no difference in MCC between huff-cough and a) HFCWO-vest; b) OPEP; and c) WBV. For hypothesis two, biomarker values pre and post each ACT intervention will be compared using paired t-tests to test the null hypothesis [i.e. there is no difference between candidate biomarker concentration before and after use of a) HFCWO-vest, b) OPEP, and c) WBV]. We will also compare the change in biomarker values across different methods using a repeated measure model. Finally, we will explore the strength of the relationship between MCC responses and the corresponding change in candidate biomarker values by calculating the Spearman's Correlation Coefficient.

Missing Data/Subject Drop Out:

Subjects that drop out before the end of visit 3 (i.e. provide only baseline data) will not be included for analysis, and will be replaced. Data from all subjects who complete at least visit 3 will be included in the analysis. Thus, partial datasets that include one of the ACT interventions will include data available allowing for the comparison of one or more ACT method to baseline. We anticipate no more than 10% drop out beyond visit 3. Per sample size estimates below, 7 subjects are estimated to provide 80% power to detect a change from baseline, so enrolling 10 subjects should provide adequate sample size for the analyses.

Sample size estimate: No comparable preliminary data exists on MCC in response to ACTs. However, our lab has collected pertinent data, i.e. the effect of 2 HS doses (given 100 minutes and 4.5 hours min after isotope was inhaled) on MCC in CF patients (N=11) using the same SILP method we propose here. The mean (SD) values at baseline and after HS are shown in Table 1. We used linear interpolation to estimate the AUC-Clr270 value for these data, as this is close to the selected final imaging time point in the proposed study.

Table 1

	Baseline, mean (SD)	Post-HS, mean (SD)	Mean Change (SD)
AUC-Clr_{180}	17.4 (11.5)	30.5 (11.6)	13.1 (9.4)
AUC-Clr_{360}	47.4 (26.6)	89.9 (27.2)	42.4 (21.2)
Interpolated AUC-Clr_{270}	32.4 (19.0)	60.2 (19.1)	27.7 (15.1)

We predict that the observed mean change in AUC-Clr_{240} after an ACT will be somewhat smaller because this study uses a baseline that likely will be accelerated by a partially active intervention (huff cough). Therefore, we estimate a more conservative predicted effect size of 20.0 (72% of that observed after HS) with the same estimate of variability (SD 15.1) in our sample size calculations. Using these data, a dependent effect size index, d_z of 1.33 was calculated for the AUC-Clr_{240} variable. Using G*Power (v3.1) and a paired, 2-tailed t-test to compare two means, we estimate that 7 subjects will be needed to detect a statistically significant difference (α = 0.05) (Figure 5). Given that we will be making multiple comparisons, wish to explore correlations between MCC and secondary outcomes, and to buffer against potential subject drop-out, we plan to enroll 10 subjects into this study.

Figure 5

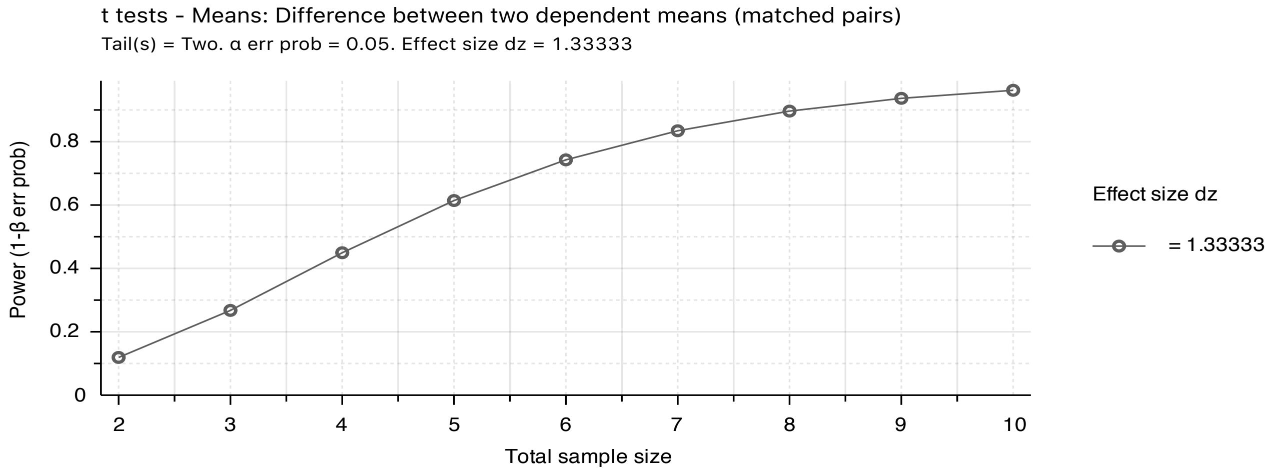
t tests - Means: Difference between two dependent means (matched pairs)
Tail(s) = Two. α err prob = 0.05. Effect size dz = 1.33333
Power (1-β err prob)
0
0.2
0.4
0.6
0.8
2
3
4
5
6
7
8
9
10
Total sample size
Effect size dz
= 1.33333

REFERENCES:

1. Boucher R. Evidence for airway surface dehydration as the initiating event in CF airway disease. Journal of internal medicine 2007;261:5-16.
2. Boucher RC. Airway surface dehydration in cystic fibrosis: pathogenesis and therapy. Annu Rev Med 2007;58:157-70.
3. Tarran R, Trout L, Donaldson SH, Boucher RC. Soluble mediators, not cilia, determine airway surface liquid volume in normal and cystic fibrosis superficial airway epithelia. The Journal of general physiology 2006;127:591-604.
4. Button B, Cai L-H, Ehre C, et al. A periciliary brush promotes the lung health by separating the mucus layer from airway epithelia. Science 2012;337:937-41.
5. Bennett W, Zeman K, Donaldson SH, Donohue JF, Knowles MR, Boucher RC. Cough Clearance is Less Effective in Cystic Fibrosis than Chronic Bronchitis. AmJRespCritCare Med 2004;169:A386.
6. Donaldson SH, Corcoran TE, Laube BL, Bennett WD. Mucociliary clearance as an outcome measure for cystic fibrosis clinical research. Proceedings of the American thoracic society 2007;4:399-405.
7. Flume PA, Robinson KA, O'Sullivan BP, et al. Cystic fibrosis pulmonary guidelines: airway clearance therapies. Respiratory care 2009;54:522-37.
8. Torvinen S, Kannus P, Sievänen H, et al. Effect of 8‐Month Vertical Whole Body Vibration on Bone, Muscle Performance, and Body Balance: A Randomized Controlled Study. Journal of bone and mineral research 2003;18:876-84.
9. Verschueren SM, Roelants M, Delecluse C, Swinnen S, Vanderschueren D, Boonen S. Effect of 6‐Month Whole Body Vibration Training on Hip Density, Muscle Strength, and Postural Control in Postmenopausal Women: A Randomized Controlled Pilot Study. Journal of bone and mineral research 2004;19:352-9.
10. B Catharine Craven MSc M. Whole-body vibration as potential intervention for people with low bone mineral density and osteoporosis: a review. Journal of rehabilitation research and development 2009;46:529.
11. Rietschel E, van Koningsbruggen S, Fricke O, Semler O, Schoenau E. Whole body vibration: a new therapeutic approach to improve muscle function in cystic fibrosis? International Journal of Rehabilitation Research 2008;31:253-6.
12. Bennett WD, Laube BL, Corcoran T, et al. Multisite comparison of mucociliary and cough clearance measures using standardized methods. Journal of aerosol medicine and pulmonary drug delivery 2013;26:157-64.
13. Donaldson SH, Bennett WD, Zeman KL, Knowles MR, Tarran R, Boucher RC. Mucus clearance and lung function in cystic fibrosis with hypertonic saline. New England Journal of Medicine 2006;354:241-50.
14. Rowe SM, Heltshe SL, Gonska T, et al. Clinical mechanism of the cystic fibrosis transmembrane conductance regulator potentiator ivacaftor in G551D-mediated cystic fibrosis. American journal of respiratory and critical care medicine 2014;190:175-84.
15. Knowles MR, Clarke LL, Boucher RC. Activation by extracellular nucleotides of chloride secretion in the airway epithelia of patients with cystic fibrosis. New England Journal of Medicine 1991;325:533-8.
16. Morse DM, Smullen JL, Davis CW. Differential effects of UTP, ATP, and adenosine on ciliary activity of human nasal epithelial cells. American Journal of Physiology-Cell Physiology 2001;280:C1485-C97.

17. Button B, Boucher RC, Group UoNCVL. Role of mechanical stress in regulating airway surface hydration and mucus clearance rates. Respiratory physiology & neurobiology 2008;163:189-201.
18. Esther CR, Olsen BM, Lin F-C, Fine J, Boucher RC. Exhaled breath condensate adenosine tracks lung function changes in cystic fibrosis. American Journal of Physiology-Lung Cellular and Molecular Physiology 2013;304:L504-L9.
19. Esther CR, Boysen G, Olsen BM, et al. Mass spectrometric analysis of biomarkers and dilution markers in exhaled breath condensate reveals elevated purines in asthma and cystic fibrosis. American Journal of Physiology-Lung Cellular and Molecular Physiology 2009;296:L987-L93.
20. Jain B, Rubinstein I, Robbins R, Leise K, Sisson JH. Modulation of airway epithelial cell ciliary beat frequency by nitric oxide. Biochemical and biophysical research communications 1993;191:83-8.
21. Li D, Shirakami G, Zhan X, Johns RA. Regulation of ciliary beat frequency by the nitric oxide–cyclic guanosine monophosphate signaling pathway in rat airway epithelial cells. American Journal of Respiratory Cell and Molecular Biology 2000;23:175-81.
22. Jain L, Chen X-J, Brown LA, Eaton DC. Nitric oxide inhibits lung sodium transport through a cGMP-mediated inhibition of epithelial cation channels. American Journal of Physiology-Lung Cellular and Molecular Physiology 1998;274:L475-L84.
23. Helms MN, Yu L, Malik B, Kleinhenz DJ, Hart CM, Eaton DC. Role of SGK1 in nitric oxide inhibition of ENaC in Na+-transporting epithelia. American Journal of Physiology-Cell Physiology 2005;289:C717-C26.
24. Grasemann H, Gonska T, Avolio J, Klingel M, Tullis E, Ratjen F. Effect of ivacaftor therapy on exhaled nitric oxide in patients with cystic fibrosis. Journal of Cystic Fibrosis 2015;14:727-32.
25. Buga GM, Gold ME, Fukuto JM, Ignarro LJ. Shear stress-induced release of nitric oxide from endothelial cells grown on beads. Hypertension 1991;17:187-93.
26. Bennett WD, Wu J, Fuller F, et al. Duration of Action of Hypertonic Saline on Mucociliary Clearance in the Normal Lung. Journal of Applied Physiology 2015:jap. 00404.2014.
27. Kempainen RR, Milla C, Dunitz J, et al. Comparison of settings used for high-frequency chest-wall compression in cystic fibrosis. Respiratory care 2010;55:695-701.
28. Esther CR, Lazaar AL, Bordonali E, Qaqish B, Boucher RC. Elevated airway purines in COPD. CHEST Journal 2011;140:954-60.
29. Patel K, Davis SD, Johnson R, Esther CR. Exhaled breath condensate purines correlate with lung function in infants and preschoolers. Pediatric pulmonology 2013;48:182-7.
