# Supplementary material for: Effect of airway clearance therapies on mucociliary clearance in adults with cystic fibrosis: A randomized controlled trial
Source: PLoS One. 2022 May 20;17(5):e0268622. doi: 10.1371/journal.pone.0268622 (PMC9122229; doi:10.1371/journal.pone.0268622)
Supplement: S5 File — (DOCX) [file pone.0268622.s006.docx]

Study Visit Structure:

At each of the study visits, the retention of inhaled particles in the chest was measured for a total of 274 minutes, during which the assigned ACT was performed during two 34-minute ACT sessions. Figure 1 shows the timeline of the study visits. Sixteen (16) minutes of imaging was performed prior to starting the first ACT session to estimate the baseline MCC rate. The two ACT sessions were separated by 146 minutes. During each 34-minute ACT session, the assigned ACT device was used in 4-minute cycles followed by a single standardized huff cough maneuver. For the baseline visit, a single huff cough maneuver was performed with each cycle. Six cycles of ACT in total, each separated by 2-minute image acquisition periods, were performed during each of the two 34-minute ACT sessions.

Fig 1. **Schematic of study procedures**.

Each study visit day was identical, with the exception of the assigned ACT that was utilized. The 34-minute ACT period is shown in greater detail with active clearance for four minutes followed by a huff-cough maneuver and a 2-minute MCC image capture every six minutes

Huff cough maneuvers were performed by inhaling to near full inspiratory capacity and then forcefully exhaling to near residual volume two times, followed by one cough. For OPEP, 10 breaths with the device at the highest setting of resistance were completed every 4-minute cycle for a total of 60 breaths. For HFCWO, the device was set on a protocol of increasing oscillatory frequency for each successive cycle as follows: 8, 9, 10, 18, 19, and 20 Hz. The first three segments were set to a device pressure setting of 10 (from the device’s arbitrary 1-10 scale), and the last three segments were set to a pressure of 6. This “Mineesota Protocol” was adopted as it commonly prescribed by CF clinics in the United States. The HFCWO device was active for 3 minutes and 45 seconds each cycle, allowing 15 seconds each cycle for device deflation, performance of huff coughing, and repositioning for image acquisition. For WBV, subjects were seated on the device with an erect back and feet flat on the floor with the device active on the high amplitude setting for 90 seconds during each cycle. The total number of coughs in all study visits (prescribed and spontaneous) were recorded by study personnel.

Gamma Scintigraphy Methods:

Prior to each study, transmission and background scans were performed to define lung borders and correct for background gamma activity(24). Placement of two fiducial markers containing Am^241^ (0.9 uCi each) were placed over the subject’s spine at approximately C7 and L1 to facilitate image registration during analysis. In order to achieve a reproducible deposition pattern that included substantial small airway deposition, the radioaerosol was inhaled using a slow (80 ml/sec), six second inhalation through a Pari LL nebulizer (PARI®, Starnberg, Germany) modiﬁed by removing the lower third of the internal bafﬂe to allow generation of large aerosol particles (MMAD 9.5 μm)(23, 25). Following inhalation of the radioaerosol, subjects were positioned in front of a gamma camera, and position was maintained with the assistance of a laser pointer aimed at a piece of tape fixed to the subject’s sternal notch. Serial images were obtained via 2-minute acquisitions.

Image analysis was performed using a standard, previously-described methodology to determine the total activity from the inhaled radiotracer within the lung region of each acquired image, corrected for background radiation and isotope decay(15). A region of interest (ROI) was drawn around the right lung, to avoid confounding activity in the stomach, which overlies the left lower lung zone. Central and peripheral ROIs were also outlined using standard methods to allow analysis of regions relatively enriched with large (central ROI) and small (peripheral ROI) airways (13). The heterogeneity of aerosol deposition was quantitatively described by calculating the skew of the whole lung signal intensity histogram using the initial deposition image. Aerosol deposition was further characterized by calculating the ratio of activity within the central versus peripheral ROIs (C/P), normalized to the ratio of counts in the central and peripheral ROIs on the transmission scan to account for variations of lung thickness within these regions(13).
