# Supplementary material for: Effect of airway clearance therapies on mucociliary clearance in adults with cystic fibrosis: A randomized controlled trial
Source: PLoS One. 2022 May 20;17(5):e0268622. doi: 10.1371/journal.pone.0268622 (PMC9122229; doi:10.1371/journal.pone.0268622)
Supplement: Attachment — Submitted filename: Authors_Trimble_Comments_PlosOne21.pdf [file pone.0268622.s008.pdf]

Comments to authors

Thank you for the opportunity for reviewing this exciting manuscript including non-conventional outcome measures to assess the effects of different ACTs at short-term in people with cystic fibrosis. I really enjoyed reading it and learned as well. However, I would like to discuss some points with the authors.

a. The timeline of the study is a bit confusing and not easy to follow-up at first glance. Could you please try to explain it in a simpler way? You chose a complex procedure and it may be difficult for future readers to follow, especially if they are not familiar with this type of outcome measures
b. Why did you assess the effects of two ACT sessions, one followed by the other? Although two sessions per day are recommended in clinical practice, they are not back-to-back.
c. What is the rationale for assessing the whole effects of ACTs only up to 40 minutes after the end of the second session? The possibility of seeing differences over a longer period has been lost.
d. I was surprised that the autogenic drainage technique was not included in the study, as it is one of the most popular techniques in this population. Why do not include techniques that increase expiratory airflow by reducing the cross-sectional ratio of the airways when they seem to be best suited according to the mucus clearance physiology?
e. I do not agree to compare the body vibration device with exercise. The main mechanism of exercise that improves airway clearance is increased airflow. However, a body vibration device could be more similar to HFCWO device.
f. Why were the pulmonary biomarkers (FeNO and EBC) only obtained after the first ACTs session? Why not also after the second ACTs session like the MCC?
g. According to table 1, six participants use daily hypertonic saline as part of their routine treatment. Did the participants take the HS the days of the study? If so, when did they take it? How do you control this factor? Could it have influenced your results?
h. Were the participants adherent/trained to any ACTs? What were the ACTs they routinely used?
i. Could you please give us more information on the daily sputum expectoration of your participants? Alternatively, any information to understand better the level of impairment of the airway clearance in your sample?

j. I believe that huff-cough manoeuvres is a good ´comparator´ in this study. However, how many standardised cough manoeuvres performed the participants in each study visit? How many spontaneous cough manoeuvres were recorded in each study visit? Were this number similar between the different treatment arms? No data were reported in results.

k. Although no difference was observed between the treatment arms; OPEP showed a tendency to clear less Tc^{99m} at the end of observed period (270 min). OPEP is the only ACTs analysed in this study that requires the active collaboration of the patient. It is described in the manuscript that the three ACTs were instructed to participants at the screening visit, however, were the participants able to test the OPEP device?; if participants were not sufficiently familiar with these devices, could lack of experience have affected your results?

l. Do you really think that FeNO is an appropriate outcome measure to assess the effects of ACTs in your sample considering that almost 25% of your measures did not achieve the minimum threshold? Please, could you explain this point in the discussion section? In addition, could you explain why there is an opposite direction of change in your study in this outcome measure?

m. I checked the information provided in the supplementary material and was surprised that you did not include any information on sputum quantity or % of solids in this manuscript. However, both outcomes seem to be assessed according to your initial protocol (supplementary material). Why did you not include this information in the manuscript?

n. Your data analysis is a bite confusing to me.

    a. First, you suggest using non-parametrical approach in methods. I agree with this approach because of your sample size and the non-normal distribution of your outcome measures. However, it seems that you use parametric tests in the results section (e.g., page 11, line 225 it is stated that you use ANOVA; it is explained in figure 4 and in page 12, line 248 that you use t-student for paired sample; you describe your results in the figures using mean instead of median). For that reason, I recommend performing again the statistical analysis using a non-parametrical approach for all your variables and describe the findings using median and P_{25}-$P_{75,}$ as you correctly did for EBC findings in table 2.

    b. Why not include the effect size calculation in the results obtained from EBC? It would be useful to understand better the magnitude of the change.

    c. It seems to me (based on your data and graphs) that you calculate correlations of two variables rather than linear regressions models. If this is correct, please change the information provided in the statistical analysis (page 10, line 198) and page 13, line 261) and give us information about the value of the correlation

(r value) with their confidence intervals at 95%. If you finally calculate linear regressions model (I do not think it is appropriate for your sample size and the distribution of your outcome measures), you need to give us information about the odds ratio and the confidence intervals of your outcome measures.

o. Table 2 shows several molecules that have not been mentioned in the introduction or in the methods section. Could you please introduce a short paragraph to explain the role of these molecules in the airway clearance and guide to the readers?

In addition, you will find my minor comments / suggestions in the following table:

**Page number**	**Sentence / Section**	**Issues which could be considered**
Page 3, line 42	Introduction	Suggest removing the word *´mechanical´* because the information is described in a general way.
Page 3, line 52	Introduction	Authors referred to a SR published in 2017; however, it is not the latest version. Could the authors please check and change the information provided according to the updated version of this SR? (Cochrane Database Syst Rev. 2020 Apr 30;4(4):CD006842.
Page 3, line 42	Introduction	Suggest removing the word *´mechanical´* because postural drainage and exercise are not theoretically considered mechanical ACTs.
Page 4, line 64	Introduction	From my point of view, the introduction section is not the best place to describe the methodology of your study. Thus, I suggest removing the information included from line 64 to the end of the paragraph. Instead of this information, the authors may i) describe the different mechanism of action of the three techniques explored in the study; ii) or even explain why the biomarkers assessed are appropriate for evaluating the effects of ACTs because they are poorly explored in this type of studies.
Page 4, line 64	Introduction	Suggest removing the word *´widely-used´* because HFCWO is not as commonly used in Europe or Asia as in the USA in this population.
Page 4, line 75	Hypothesis	Please, clarify that MCC/CC was your primary endpoint and the rest of outcome measures were considered secondary endpoints.
Page 5, line 86	Methods	How long was the washout period between the treatment arms? Was the washout period standardised?

Page 7, line 130	Methods	Why did the authors increase the frequency each cycle? Why did the authors decide to set different pressure in each cycle? What is the reason for using this protocol?
Page 7, line 138	Methods	Please, clarify that the number of coughs were registered in all clinic visits and not only in the last one.
Page 10, table 1	Results	Did the participants have a chronic airway infection? Please, include additional information on possible pathogens in table 1
Page 12, fig 3 (B); fig 4	Results	Please, change ´Vest´ to HFCWO
Page 14, line 276	Discussion	Suggest including at short-term at the end of the sentence because you did not explore longer-term effects (24h for example).
Page 14, line 292	Discussion	Suggest being caution with this sentence. In your study, you did not find any difference between ACTs plus huff-cough and huff-cough alone **at very short-term** and did not evaluate the most popular ACTs in this population**.** This does not mean that the techniques may not provide any clinical benefit in this population
Page 15, line 300	Discussion	The limitation described for MCC is similar to lung clearance index to assess the effects of ACTs. Could you describe/name it?
Page 16, line 318	Discussion	Suggest including your small sample size as a limitation to find a more reliable results in purines and the other small molecules.
