# Supplementary material for: Effect of airway clearance therapies on mucociliary clearance in adults with cystic fibrosis: A randomized controlled trial
Source: PLoS One. 2022 May 20;17(5):e0268622. doi: 10.1371/journal.pone.0268622 (PMC9122229; doi:10.1371/journal.pone.0268622)
Supplement: Attachment — Submitted filename: Editor_Trimble_Comments_PlosOne21.pdf [file pone.0268622.s009.pdf]

Comments to editors

Dear Michelle Dohm,

It was a pleasure to review this manuscript entitled "Effect of Airway Clearance Therapies on Mucociliary Clearance in Adults with Cystic Fibrosis: a randomized controlled trial" (PONE-D-21-05105), thus I really appreciate this opportunity. Sorry for my delay but I have done a deep review of the manuscript.

Taking into account all the comments/suggestion described in the attached file, the manuscript is not ready for publication yet. My major comments related to this manuscript are:

a. Data analysis approach seems to be confusing. Authors described different statistical test in methods and results section. Therefore, it is important that authors clarify this point.
b. Authors need to provide more information about the baseline characteristics of the participants related to airway clearance. This manuscript included only ten participants and, although all of them have a diagnosis of CF, this does not mean that they have significant mucociliary transport impairment. In fact, three of them have normal lung function according to FEV_1 values.
c. Authors used non-conventional outcome measures to assess the effects of ACTs. The findings suggest that the addition of any ACTs to huff-cough has no impact on airway clearance. However, the small sample size, the psychometric properties of these outcome measures (low reliability, high variability between participants) or even the ability to detect change after a very short-term period could be also reason for the negative results. Therefore, I think it is important to highlight these limitations as well.
d. The manuscript is well written, but the authors chose a complex procedure and it may be difficult for future readers to follow, especially if they are not familiar with this type of outcome measures. For that reason, I think the authors should simplify it and always follow the same order of description throughout the manuscript.

Sincerely

Beatriz Herrero-Cortina
