# Supplementary material for: Effect of airway clearance therapies on mucociliary clearance in adults with cystic fibrosis: A randomized controlled trial
Source: PLoS One. 2022 May 20;17(5):e0268622. doi: 10.1371/journal.pone.0268622 (PMC9122229; doi:10.1371/journal.pone.0268622)
Supplement: Attachment — Submitted filename: Reviewers_comments.docx [file pone.0268622.s010.docx]

**Comments to the Author**

1. Is the manuscript technically sound, and do the data support the conclusions?

The manuscript must describe a technically sound piece of scientific research with data that supports the conclusions. Experiments must have been conducted rigorously, with appropriate controls, replication, and sample sizes. The conclusions must be drawn appropriately based on the data presented.

Reviewer #1: Yes

Reviewer #2: Yes

Reviewer #3: Yes

2. Has the statistical analysis been performed appropriately and rigorously?

Reviewer #1: No

Reviewer #2: No

Reviewer #3: Yes

3. Have the authors made all data underlying the findings in their manuscript fully available?

The [PLOS Data policy](http://www.plosone.org/static/policies.action#sharing) requires authors to make all data underlying the findings described in their manuscript fully available without restriction, with rare exception (please refer to the Data Availability Statement in the manuscript PDF file). The data should be provided as part of the manuscript or its supporting information, or deposited to a public repository. For example, in addition to summary statistics, the data points behind means, medians and variance measures should be available. If there are restrictions on publicly sharing data—e.g. participant privacy or use of data from a third party—those must be specified.

Reviewer #1: Yes

Reviewer #2: No

Reviewer #3: Yes

4. Is the manuscript presented in an intelligible fashion and written in standard English?

PLOS ONE does not copyedit accepted manuscripts, so the language in submitted articles must be clear, correct, and unambiguous. Any typographical or grammatical errors should be corrected at revision, so please note any specific errors here.

Reviewer #1: Yes

Reviewer #2: Yes

Reviewer #3: Yes

5. Review Comments to the Author

Please use the space provided to explain your answers to the questions above. You may also include additional comments for the author, including concerns about dual publication, research ethics, or publication ethics. (Please upload your review as an attachment if it exceeds 20,000 characters)

Reviewer #1: The objective of this study is to investigate the effectiveness of airway clearance therapy on mucociliary clearance (MCC) adults with cystic fibrosis (CF). The authors considered an open-label, multiple cross-over study study. The study was approved by the Institutional Review Board at Univ. of North Carolina, and carries a legitimate NCT number. While the study objectives sound interesting, a number of shortcomings were observed, in regards to abiding by the CONSORT guidelines for conducting and reporting results of high-quality randomized clinical trials (RCTs), as well as some statistical and reporting issues:

1. Abstract:
It's better to present the Abstract in accordance to the 4 sections: Background, Methods, Results and Conclusions. Also, in the Results section, statistical significance/non-significance of findings should be expressed with p-values, and estimated precision, such as confidence intervals, or CIs (say, 95\%). Check CONSORT checklist for Abstracts reporting of RCTs.

*We appreciate the reviewer’s advice. The abstract has now been restructured. References to significance have been removed, using only p-values, confidence intervals, etc to allow readers to draw their own conclusions on significance.*

2. Methods:

Methods reporting appeared very messy. An orderly manner is suggested, following CONSORT guidelines, without repeating information, such as Trial Design, Participant Eligibility Criteria and settings, Interventions, Outcomes, sample size/power considerations, Interim analysis and stopping rules, Randomization (details on random number generation, allocation concealment, implementation), Blinding issues, etc. The authors are advised to create separate subsections for each of the possible topics (whichever necessary), and that way produce a very clear writeup. I see the Authors indeed made an attempt; however, they are advised to write it carefully, following nice examples in the manuscript below:

<https://www.sciencedirect.com/science/article/pii/S0889540619300010>

*Methods section has been completely restructured using the headings suggested. In order to address this important point (as well as similar points regarding clarity from other reviewers), some specific details on the timing of study procedures and technical aspects of gamma scintigraphy have been moved to supplementary material to keep the manuscript manageable and concise.*

(a) For instance, the randomization technique employed (Latin square method) looks almost hidden; it should be pointed clearly. Furthermore, randomization and allocation concealment should be made very clear; the trial staff recruiting patients should not have the randomization list. Randomization should be prepared by the trial statistician, and he/she would not participate in the recruiting.

*In response to this important point, randomization methods have been given its own section. Note, randomization sequence was performed by the statistician, but was not concealed from other study personnel as this was an unblinded study. Given the nature of the study, particularly with a crossover design with ample washout between visits (now included and highlighted), we do not see biological plausibility for the lack of concealment as means for the introduction of meaningful bias.*

(b) I am surprised to see no statement on sample size/power in a manuscript proposing a cross-over trial. A vague statement was provided as "Sample size was determined based on..", with no specific determination of sample size wrt. some desired effect size. This is really a key here; else one has no idea why the study recruited and analyzed only 10 subjects.

*Thank you for pointing this out. We have added the* a priori *sample size calculation/estimate contained in our protocol in our methods section.*

(c) Friedman's one-way nonparametric ANOVA test was used, which looks OK. However, to assess correlation between changes in candidate biomarkers, the authors stated a linear regression was used. From my understanding of cross-over trials, a repeated measures analysis, via linear mixed-models is more appropriate. In that context, it is doubtful how much credible the study findings will be with only 10 subjects!

*We concur with this critique on the use of linear regressions in this manner for this study. However, given that it was our intent to consider each small molecule independently in relationship with MCC (and not as a single model taking into account each candidate biomarker simultaneously), as well as our limited sample size, we don’t believe a linear mixed model would be appropriate here. However, we have re-analyzed the data using Spearman correlation between change in concentration and Ave274Clr, which we believe is a more appropriate method for investigating the correlation between these biomarker levels and MCC. None of these achieved a p-value that we considered significant individually, even without adjusting for multiple comparisons. However, Spearman’s correlation with 95% CI and p-values have been added to table 2 for completeness. Thus, our conclusions regarding an identified relationship between adenosine and MCC have been removed.*

3. Results & Conclusions:
The authors ignored a thoughtful discussion of the presence/absence of a desirable 'washout' period, which is the cornerstone of a cross-over trial. The sample size enrolled is somewhat small, reflecting a specific population. I can only consider this study, at best, as a very pilot study. However, with no clear description of how they came up with 10 subjects, I am hesitant to proceed further.

For understanding and designing a nice multi-crossover study, the authors are asked to follow this article below (which also discusses the utility of using random-effects models, or the linear/nonlinear mixed-effects models):

<https://www.ncbi.nlm.nih.gov/pmc/articles/PMC6481185/>

*Thank you for these comments. We have included information on the washout period in this study. We have expounded on the limitations of our study, including noting its sample size. Given extremely low plausibility for biological of effects from each intervention in this study lasting beyond 24 hours, we remain confident in our relatively simple crossover study design.*

Reviewer #2: Thank you for the opportunity for reviewing this exciting manuscript including non-conventional outcome measures to assess the effects of different ACTs at short-term in people with cystic fibrosis. I really enjoyed reading it and learned as well. However, I would like to discuss some points with the authors.

a. The timeline of the study is a bit confusing and not easy to follow-up at first glance. Could you please try to explain it in a simpler way? You chose a complex procedure and it may be difficult for future readers to follow, especially if they are not familiar with this type of outcome measures

*Thank you for this comment. We have simplified the methods section and moved more of the complex technical details into a supplement.*

b. Why did you assess the effects of two ACT sessions, one followed by the other? Although two sessions per day are recommended in clinical practice, they are not back-to-back.

*We used two ACT sessions (separated by ~2 hours) after a single inhalation of radiotracer in an attempt to increase the likelihood of capturing an ACT effect on MCC and to potentially distinguish the magnitude of effect between ACT methods. As we did not know what to expect with respect to the size or duration of an ACT effect on MCC, we hoped that by measuring MCC both after a single ACT session and after a second session, we might reduce our risk of missing an important effect.*

c. What is the rationale for assessing the whole effects of ACTs only up to 40 minutes after the end of the second session? The possibility of seeing differences over a longer period has been lost.

*The methodology of gamma scintigraphy has used a 90-minute session as standard timeframe for image acquisition; particularly since subjects must sit still with minimal movements, this is approaching the upper limit for what is feasible. We inserted a ~30-minute ACT session early into this 90-minute session to allow for reasonable measurement of MCC before, during, and after ACT. The second period of image acquisition ~2 hours later provided a means of detecting later effects.*

d. I was surprised that the autogenic drainage technique was not included in the study, as it is one of the most popular techniques in this population. Why do not include techniques that increase expiratory airflow by reducing the cross-sectional ratio of the airways when they seem to be best suited according to the mucus clearance physiology?

*During study design, we were greatly concerned with ensuring that study procedures could be carefully standardized across all subjects as were familiar and applicable to our population. Patients in the United States are much less familiar with autogenic drainage, and we, therefore, worried about our ability to standardize this technique across all subjects. Further, we felt that performance of 4 study arms was the maximum that could feasibly be performed for multiple reasons, and therefore had to choose between several possible ACT interventions while also driving the decision to compare mechanical device methods to huff coughing rather than a true baseline MCC measurement.*

e. I do not agree to compare the body vibration device with exercise. The main mechanism of exercise that improves airway clearance is increased airflow. However, a body vibration device could be more similar to HFCWO device.

*We have clarified the language to state that the whole-body vibration device was developed for the purposes to enhance exercise, not as exercise itself.*

f. Why were the pulmonary biomarkers (FeNO and EBC) only obtained after the first ACTs session? Why not also after the second ACTs session like the MCC?

*We felt that these biomarkers, if impacted by ACT, would change acutely (hence comparison between baseline and first ACT treatment). This initial change would likely confound any subsequent measurements that followed (i.e. with second ACT), given uncertainty regarding how quickly they would return to baseline. Further, the cost associated with the assays for these biomarkers was an additional consideration in our study design.*

g. According to table 1, six participants use daily hypertonic saline as part of their routine treatment. Did the participants take the HS the days of the study? If so, when did they take it? How do you control this factor? Could it have influenced your results?

*We have clarified in the methods section that participants held their use of mucolytics and home ACT prior to study visit days. It is possible that regular use (or not) of these agents in a timeframe beyond that had an effect on our results, however, we do not believe this was substantial enough to have significantly changed our final results.*

h. Were the participants adherent/trained to any ACTs? What were the ACTs they routinely used?

*We clarified in the methods section that all subjects were trained on proper technique of the ACTs. We did not collect data on adherence on home OPEP/HFCWO, but agree that such information this would have been helpful to collect.*

i. Could you please give us more information on the daily sputum expectoration of your participants? Alternatively, any information to understand better the level of impairment of the airway clearance in your sample?

*We did not collect information on daily sputum production, in part due to the subjective nature of this assessment. We used lung function expressed by FEV1 as a measure of both airflow limitation and likelihood of impaired mucus clearance.*

j. I believe that huff-cough manoeuvres is a good ´comparator´ in this study. However, how many standardised cough manoeuvres performed the participants in each study visit? How many spontaneous cough manoeuvres were recorded in each study visit? Were this number similar between the different treatment arms? No data were reported in results.

*We clarified in our methods (now moved into supplemental material) that six huff-coughs were performed during for each ACT session. Data for spontaneous coughs is included in the supplemental material (in the MCC excel spreadsheet). The number of total coughs were similar across all arms.*

k. Although no difference was observed between the treatment arms; OPEP showed a tendency to clear less Tc99m at the end of observed period (270 min). OPEP is the only ACTs analysed in this study that requires the active collaboration of the patient. It is described in the manuscript that the three ACTs were instructed to participants at the screening visit, however, were the participants able to test the OPEP device?; if participants were not sufficiently familiar with these devices, could lack of experience have affected your results?

*As mentioned above, all subjects were trained on proper technique at the screening visit. This has been clarified in the methods section. Since the effectiveness of the device is effort-dependent, it is possible that improper use across several of the subjects led to our negative findings. However, we do not see a way of assessing for (much less controlling for) this beyond our methods of teaching and observing technique. Importantly, we did not observe difficulties with performance of the OPEP technique in any of our study subjects.*

l. Do you really think that FeNO is an appropriate outcome measure to assess the effects of ACTs in your sample considering that almost 25% of your measures did not achieve the minimum threshold? Please, could you explain this point in the discussion section? In addition, could you explain why there is an opposite direction of change in your study in this outcome measure?

*Our experience with FeNO and the specific device used for the study was limited, and was included as a secondary outcome. While we were anticipating that many patients would have lower-than-normal FeNO values, we did not anticipate that several subjects would fall outside the detectable limit of detection. Because we anticipated that ACTs would, if anything, increase FeNO (as a marker of increased ciliary activity), a low baseline measurement was not necessarily concerning. While we cannot definitively explain why our hypothesis was wrong with regard to FeNO, we have updated the discussion to include our best conjecture as to why we observed what we did.*

m. I checked the information provided in the supplementary material and was surprised that you did not include any information on sputum quantity or % of solids in this manuscript. However, both outcomes seem to be assessed according to your initial protocol (supplementary material). Why did you not include this information in the manuscript?

*We have updated the methods and results section to report our data from sputum collection. There were too few data to perform quantitative analysis. Individual results are included in supplementary material.*

n. Your data analysis is a bite confusing to me.

a. First, you suggest using non-parametrical approach in methods. I agree with this approach because of your sample size and the non-normal distribution of your outcome measures. However, it seems that you use parametric tests in the results section (e.g., page 11, line 225 it is stated that you use ANOVA; it is explained in figure 4 and in page 12, line 248 that you use t-student for paired sample; you describe your results in the figures using mean instead of median). For that reason, I recommend performing again the statistical analysis using a non-parametrical approach for all your variables and describe the findings using median and P25-P75, as you correctly did for EBC findings in table 2.

*We have clarified the results section to indicate we used Friedman's one-way nonparametric ANOVA. As for FeNO results, while it was true that these data were normal and a t-test could have been appropriate, we agree that mixing parametric and non-parametric methods is confusing at best. For consistency’s sake, we have re-analyzed the data using Wilcoxon signed-rank test, as was used elsewhere in the study. We have updated the figure of FeNO data to indicate median and P25-75.*

b. Why not include the effect size calculation in the results obtained from EBC? It would be useful to understand better the magnitude of the change.

*We appreciate this suggestion, and did spend some time performing effect size calculations, but ultimately believe that including these add more obfuscation than insight to a data-heavy section of our results. The appropriate effect size calculation for the analysis used (Wilcoxon) is appears to be a rather obscure measure (Matched Pairs Rank biserial r), and is likely to have little meaning to readers of this paper. Given the measures from EBC are indices (relative concentrations normalized to urea), and there was one value whose value substantially changed (Lactate), inclusion of this effect size calculation seems to add little value to the results.*

c. It seems to me (based on your data and graphs) that you calculate correlations of two variables rather than linear regressions models. If this is correct, please change the information provided in the statistical analysis (page 10, line 198) and page 13, line 261) and give us information about the value of the correlation (r value) with their confidence intervals at 95%. If you finally calculate linear regressions model (I do not think it is appropriate for your sample size and the distribution of your outcome measures), you need to give us information about the odds ratio and the confidence intervals of your outcome measures.

*We concur with this critique on the appropriateness of correlations over use of linear regression models. We did change our analysis from linear regression to Spearman correlation calculation. The previously identified relationship did not hold using this method, and instead, all Spearman correlation coefficients (with 95% CI and p-values) are now included.*

o. Table 2 shows several molecules that have not been mentioned in the introduction or in the methods section. Could you please introduce a short paragraph to explain the role of these molecules in the airway clearance and guide to the readers?

*Thank you for this suggestion. We have updated the introduction to briefly introduce these molecules by class.*

In addition, you will find my minor comments / suggestions in the following table:

Page number Sentence / Section Issues which could be considered:

Page 3, line 42 Introduction Suggest removing the word ´mechanical´ because the information is described in a general way.

- *We have removed the word “mechanical”*

Page 3, line 52 Introduction Authors referred to a SR published in 2017; however, it is not the latest version. Could the authors please check and change the information provided according to the updated version of this SR? (Cochrane Database Syst Rev. 2020 Apr 30;4(4):CD006842.

- *Reference updated*

Page 3, line 42 Introduction Suggest removing the word ´mechanical´ because postural drainage and exercise are not theoretically considered mechanical ACTs.

- *We have removed the word “mechanical”*

Page 4, line 64 Introduction From my point of view, the introduction section is not the best place to describe the methodology of your study. Thus, I suggest removing the information included from line 64 to the end of the paragraph. Instead of this information, the authors may i) describe the different mechanism of action of the three techniques explored in the study; ii) or even explain why the biomarkers assessed are appropriate for evaluating the effects of ACTs because they are poorly explored in this type of studies.

- *We have updated this section of the introduction for simplicity.*

Page 4, line 64 Introduction Suggest removing the word ´widely-used´ because HFCWO is not as commonly used in Europe or Asia as in the USA in this population.

- *We have updated language to note the regional differences in ACT techniques.*

Page 4, line 75 Hypothesis Please, clarify that MCC/CC was your primary endpoint and the rest of outcome measures were considered secondary endpoints.

- *We now plainly state our primary and secondary hypotheses and endpoints.*

Page 5, line 86 Methods How long was the washout period between the treatment arms? Was the washout period standardised?

- *Yes, this was standardized and in our protocol. We have included information in the methods section on washout.*

Page 7, line 130 Methods Why did the authors increase the frequency each cycle? Why did the authors decide to set different pressure in each cycle? What is the reason for using this protocol?

- *This is the most common standardized HFCWO protocol used in the US and is frequently prescribed to patients. This information on HFCWO settings has been moved to supplemental material, but now reflects this as the often-termed “Minnesota Protocol.”*

Page 7, line 138 Methods Please, clarify that the number of coughs were registered in all clinic visits and not only in the last one.

- *Manuscript updated to clarify coughing counts at all visits (now in supplementary material).*

Page 10, table 1 Results Did the participants have a chronic airway infection? Please, include additional information on possible pathogens in table 1

- *We did not collect clinical information on chronic infection. Although this is a reasonable consideration, we do not believe the presence of this information would have helped identify any clearer explanation for our findings.*

Page 12, fig 3 (B); fig 4 Results Please, change ´Vest´ to HFCWO

- *Figure updated to “HFCWO” for consistency.*

Page 14, line 276 Discussion Suggest including at short-term at the end of the sentence because you did not explore longer-term effects (24h for example).

- *Included phrase “short term” – agree with reviewer’s comment.*

Page 14, line 292 Discussion Suggest being caution with this sentence. In your study, you did not find any difference between ACTs plus huff-cough and huff-cough alone at very short-term and did not evaluate the most popular ACTs in this population. This does not mean that the techniques may not provide any clinical benefit in this population

- *We elected to remove this sentence – it seems highly unlikely that ACT has no effect, but feel the responsibility to justify our findings.*

Page 15, line 300 Discussion The limitation described for MCC is similar to lung clearance index to assess the effects of ACTs. Could you describe/name it?

- *While we appreciate the comparison to LCI/MBW, for simplicity, we elected to not reference a completely different modality (which measures ventilation inhomogeneity, rather than mucus transport), which would likely require a significant degree of explanation just to speculate, which we believe is beyond the scope of this manuscript.*

Page 16, line 318 Discussion Suggest including your small sample size as a limitation to find a more reliable results in purines and the other small molecules.

- *We have included the limitation of sample size for our exploratory secondary endpoints.*

Reviewer #2 Comments to Editors -

a) Data analysis approach seems to be confusing. Authors described different statistical test in methods and results section. Therefore, it is important that authors clarify this point.

*As indicated above, we have simplified our data analysis for consistency, namely moving to all non-parametric methods as well as changing analysis from linear regression to correlation analysis.*

b) Authors need to provide more information about the baseline characteristics of the participants related to airway clearance. This manuscript included only ten participants and, although all of them have a diagnosis of CF, this does not mean that they have significant mucociliary transport impairment. In fact, three of them have normal lung function according to FEV 1 values.

*Interestingly, our extensive prior work does not reveal a relationship between FEV1 and whole lung MCC. Further, the work of others in CF infants suggests that mucus retention (i.e. failed clearance) is an early/primary defect (Esther et al Sci Transl Med. 2019 Apr 3) that results from CFTR dysfunction (Hill et al. Eur Respir J. 2018 Dec 6;52(6); Xie et al, Ann Am Thorac Soc. 2018 Nov 15). Further, because the specific modality of ACT use at home is unlikely to “carry over” to impact what we observed in study visits, full detailing of ACT methods at baseline probably has little additional value.*

c) Authors used non-conventional outcome measures to assess the effects of ACTs. The findings suggest that the addition of any ACTs to huff-cough has no impact on airway clearance. However, the small sample size, the psychometric properties of these outcome measures (low reliability, high variability between participants) or even the ability to detect change after a very short-term period could be also reason for the negative results. Therefore, I think it is important to highlight these limitations as well.

*Because the MCC and secondary outcomes we measured are fully quantitative, we are not sure what “psychometric properties” the reviewer is referring to that would impact our results. Because we used a cross-over design, intra-subject repeatability, rather than between participant variability, is in fact a much more important issue for our endpoints. In unpublished data, intrasubject repeatability for MCC is fairly high (R ~0.7 for paired measures 2 weeks apart in similar CF population), suggesting that intrasubject repeatability is reasonably good for MCC. We agree, however, that the short-term result we assessed is a potentially important issue, and we have made significant efforts to outline the multiple potential limitations that could explain or negative findings.*

d) The manuscript is well written, but the authors chose a complex procedure and it may be difficult for future readers to follow, especially if they are not familiar with this type of outcome measures. For that reason, I think the authors should simplify it and always follow the same order of description throughout the manuscript.

*We concur with this critique and have simplified our methods where possible, including moving some of the technical descriptions to supplementary material.*

Reviewer #3: Comments to Author:
Trimble and colleagues present a well-written paper addressing differences in airway clearance therapies on mucociliary clearance in people with CF. Although a similar study has been previously conducted with a similar sample size, the authors were able to rigorously study their research question in a well-designed study and include more current therapies and devices. Additionally, the authors were able to incorporate a novel secondary aim of evaluating small molecules as potential biomarkers of ACT clearance. Although the results of the study were negative, the question remains important and a topic for future research given widespread use of CFTR modulators and the need for individualized ACT therapies based on patient preference and cost effectiveness.

Title: No changes to suggest

Abstract: Incorporates main points concisely, no major changes to suggest

Intro:
The authors nicely provide relevant background while stating the primary objective of assessing for efficacy of ACT methods on mucociliary clearance.

P3, line 57) If word limit allows, would consider adding a sentence on how gamma scintigraphy works given the wider range of the PLOS audience (ie: technique that combines administration of radioistopes in conjunction with imaging modalities, etc).

*We have summarized the technique of MCC/gamma scintigraphy and have moved more of the technical details into a supplement.*

Methods:
P5, line 85-86) Would ask authors to consider further describing timing of study visits, mentions 4 study visits but the timing of visits is not understood

*We have updated the manuscript to describe timing of study visits with washout periods (between 3 and 21 days).*

Results:
P10, line 205) Would authors be able to elaborate on the number of participants/readings the FENO device malfunctioned? **It is found further down in results which would be fine to leave it placed there

*We have removed the initial sentence in the results section on the lack of reliable FeNO device for clarity.*

Discussion:
Authors could consider commenting on small sample size of study, as it was similar to the prior study referenced in their article (ref 18). Otherwise, authors appropriately discuss limitations, notably obtaining a true baseline as the huffing group may have impacted their results. Given this study was performed prior to widespread use of CFTR modulators, it would be interesting to also include exercise as a group in future studies given that many people with CF have self-discontinued use of some ACT devices given overall improvement in clinical health.

*We have updated the manuscript to comment on our sample size. Exercise is an interesting consideration for future study – and the current impact of CFTR modulators on MCC and ACT efficacy (including exercise) is indeed an important future consideration.*

6. PLOS authors have the option to publish the peer review history of their article ([what does this mean?](https://journals.plos.org/plosone/s/editorial-and-peer-review-process#loc-peer-review-history)). If published, this will include your full peer review and any attached files.


If you choose “no”, your identity will remain anonymous but your review may still be made public.


**Do you want your identity to be public for this peer review?** For information about this choice, including consent withdrawal, please see our [Privacy Policy](https://www.plos.org/privacy-policy).

Reviewer #1: No

Reviewer #2: **Yes:** Beatriz Herrero-Cortina

Reviewer #3: No
